# Vitellogenin Receptor (VgR) Mediates Oocyte Maturation and Ovarian Development in the Pacific White Shrimp (*Litopenaeus vannamei*)

**DOI:** 10.3389/fphys.2020.00485

**Published:** 2020-05-15

**Authors:** Yao Ruan, Nai-Kei Wong, Xin Zhang, Chunhua Zhu, Xiaofen Wu, Chunhua Ren, Peng Luo, Xiao Jiang, Jiatai Ji, Xugan Wu, Chaoqun Hu, Ting Chen

**Affiliations:** ^1^CAS Key Laboratory of Tropical Marine Bio-resources and Ecology (LMB), Guangdong Provincial Key Laboratory of Applied Marine Biology (LAMB), South China Sea Institute of Oceanology, Chinese Academy of Sciences, Guangzhou, China; ^2^University of Chinese Academy of Sciences, Beijing, China; ^3^National Clinical Research Center for Infectious Diseases, Shenzhen Third People’s Hospital, The Second Hospital Affiliated to Southern University of Science and Technology, Shenzhen, China; ^4^College of Fisheries, Guangdong Ocean University, Zhanjiang, China; ^5^Institution of South China Sea Ecology and Environmental Engineering (ISEE), Chinese Academy of Sciences, Guangzhou, China; ^6^Guangdong Haimao Investment Co., Ltd., Zhanjiang, China; ^7^Key Laboratory of Freshwater Aquatic Genetic Resources, Ministry of Agriculture, Shanghai Ocean University, Shanghai, China

**Keywords:** vitellogenin receptor, crustacean, phylogenetic analysis, vitellogenesis, mRNA expression, ovarian development, oocyte maturation

## Abstract

Oocyte maturation and ovarian development are sequentially coordinated events critical to reproduction. In the ovaries of adult oviparous animals such as birds, bony fish, insects, and crustaceans, vitellogenin receptor (VgR) is a plasma membrane receptor that specifically mediates vitellogenin (Vg) transport into oocytes. Accumulation of Vg drives sexual maturation of the female crustaceans by acting as a pivotal regulator of nutritional accumulation within oocytes, a process known as vitellogenesis. However, the mechanisms by which VgR mediates vitellogenesis are still not fully understood. In this study, we first identified a unique *VgR* (*Lv-VgR*) and characterized its genomic organization and protein structural domains in *Litopenaeus vannamei*, a predominant cultured shrimp species worldwide. This newly identified *Lv-VgR* phylogenetically forms a group with VgRs from other crustacean species within the arthropod cluster. Duplicated LBD/EGFD regions are found exclusively among arthropod VgRs but not in paralogs from vertebrates and nematodes. In terms of expression patterns, *Lv-VgR* transcripts are specifically expressed in ovaries of female shrimps, which increases progressively during ovarian development, and rapidly declines toward embryonic development. The cellular and subcellular locations were For analyzed by *in situ* hybridization and immunofluorescence, respectively. The *Lv-VgR* mRNA was found to be expressed in the oocytes of ovaries, and Lv-VgR protein was found to localize in the cell membrane of maturing oocytes while accumulation of the ligand Vg protein assumed an even cytoplasmic distribution. Silencing of *VgR* transcript expression by RNAi was effective for stunting ovarian development. This present study has thus provided new insights into the regulatory roles of *VgR* in crustacean ovarian development.

## Introduction

Ovarian development of oviparous animal comprises the stages of oogonia, previtellogenesis and vitellogenesis (Charniauxcotton, 1985). During development of oocytes, vitellogenesis occurs as a key process for accumulation of proteins, fats and other nutrients. Unlike the case of viviparous animals, embryos of oviparous animals develop and mature independently outside the parental body, implying that most of the developmentally requisite nutrients need to be supplied from the yolk. The quality of yolk is consequently an important factor in the successful reproduction of oviparous animals (Parma et al., 2015). Evidently, vitellogenesis is necessary for oocyte development in oviparous animals, as a critical preparatory stage to build up energy reserves and biomolecules for subsequent embryonic development (Tsukimura, 2001).

In crustaceans, the size of oocytes expands rapidly by several hundred times after the inception of vitellogenesis, with a mass of nutrients accumulated (Chen et al., 2018a). Vitellin is the most abundant protein component of yolk, which constitutes 60–90% of the total yolk protein in the eggs of crustaceans (Eastman-Reks and Fingerman, 1985). Vitellogenin (Vg) is a protein precursor of vitellin, and it is considered to be synthesized, processed, transported, absorbed and finally forms the yolk (Wahli et al., 1981). Interestingly, the sites of Vg synthesis are reported to be both endogenous and exogenous, which vary among different species, for example: the intestine of nematodes (Kimble and Sharrock, 1983), liver of fish (Hiramatsu et al., 2015), fat body of insects (Hagedorn and Fallon, 1973), ovary of sea urchins (Yokota et al., 2003), and oysters (Ni et al., 2014), and ovary and/or hepatopancreas in crustaceans (Tsutsui et al., 2000; Okuno et al., 2002; Tseng et al., 2002; Tsang et al., 2003; Mak et al., 2005; Jia et al., 2013; Chen et al., 2018b), etc. In the case of exogenous Vg, the protein is transported through blood or hemolymph and absorbed into the oocytes through endocytosis mediated by vitellogenin receptor (VgR) (Li et al., 2001; Warrier and Subramoniam, 2002).

The process of Vg/VgR endocytosis has been mainly demonstrated in oviparous amphibians (Wall and Patel, 1987), fish (Hara et al., 2016), and insects (Sappington and Raikhel, 1998). In general, exogenous Vg in the circulation binds to VgR located at the plasma membrane of oocytes to form a complex (Wall and Patel, 1987). The Vg/VgR complex is internalized into the cytoplasm as coated vesicles (Sappington and Raikhel, 1998). Finally, Vg is processed to become the mature yolk protein, while VgR is recruited to the cell membrane through the tubular vesicles (Sappington and Raikhel, 1998). Thus, VgR plays an essential role in the ovarian development of oviparous animals, which is further supported by evidence that genetic ablation or mutations of VgR could result in impaired or abnormal ovarian development, and even female sterility in birds (Bujo et al., 1995) and insects (Lin et al., 2013).

Vitellogenin receptor belongs to the low-density lipoprotein receptor (LDLR) family (Hussain et al., 1999). Structurally, it consists of five highly conserved domains, namely, the ligand-binding domain (LBD), EGF-precursor homology domain (EGFD), *O*-liked sugar domain (OLSD), transmembrane domain (TM) and cytosolic domain that contains an internalization motif (IM) (Tufail and Takeda, 2009). VgR cDNAs have been amply found in oviparous invertebrates and lower vertebrates, including birds (Stifani et al., 1990), fish (Tao et al., 1996; Hiramatsu et al., 2004; Mizuta et al., 2013), amphibians (Okabayashi et al., 1996), arthropod insects (Schonbaum et al., 1995; Lin et al., 2013; Zhang H. et al., 2019) and crustaceans (Tiu et al., 2008; Roth and Khalaila, 2012; Bai et al., 2016). In crustaceans including *Penaeus monodon* (Tiu et al., 2008), *Macrobrachium nipponense* (Bai et al., 2016), and *Macrobrachium rosenbergii* (Roth and Khalaila, 2012; Guo et al., 2019), transcript expression of *VgR* is specifically detected in the ovaries, with the maximal expression levels being reached during the mid-ovarian developmental stages. RNA interference (RNAi) of *VgR* reportedly suppressed Vg accumulation in the ovary of *P. monodon* (Tiu et al., 2008) and delayed the maturation of the ovary in *M. nipponense* (Bai et al., 2016). However, the mechanistic details of VgR regulation of ovarian development is still incompletely understood and at times shown to be controversial in different crustacean species (Tiu et al., 2008; Bai et al., 2016).

The Pacific white shrimp (*Litopenaeus vannamei*), belonging to Arthropoda, Crustacea, and Decapoda in taxonomy, is the most important cultured shrimp species in the world. In captivity, the ovaries of cultured *L. vannamei* are often unable to mature as they would naturally (Chen et al., 2014). Following artificial unilateral eyestalk ablation and nutritional supplementation, however, the ovaries of *L. vannamei* can mature and allow spawning within 3–5 days (Chen et al., 2018a). In *L. vannamei*, the precise source of Vg production during vitellogenesis remains obscure, for which the ovary as an endogenous site and the hepatopancreas as an exogenous site have both been suggested (Raviv et al., 2006; Ventura-Lopez et al., 2017). The regulatory mechanisms for *Vg* gene expression have also been explored in the ovary (Tsutsui et al., 2007, 2013; Bae et al., 2017; Chen H. Y. et al., 2018; Kang et al., 2019) and hepatopancreas (Chen et al., 2014, 2018a,b; Luo et al., 2015). In addition to Vg synthesis, efficient absorption and accumulation of Vg by the oocytes in the ovary is another vital process for vitellogenesis in oviparous animal (Stifani et al., 1990). However, information regarding how VgR-mediated Vg accumulation and ovarian development is achieved in *L. vannamei* is still limited. In this study, we established the genetic basis and functional importance of VgR in *L. vannamei* ovarian maturation by (1) identifying the full-length cDNA of *VgR* in *L. vannamei* (*Lv-VgR*) and analyzing its corresponding gene and protein structures; (2) determining the expression patterns of *Lv-VgR* in different tissues, across ovarian developmental stages including embryonic and larval periods; (3) visualizing the *Lv-VgR* mRNA and protein positive cells in the ovaries; and (4) evaluating the effects of *Lv-VgR* RNAi on the ovarian development in morphological, anatomical and histological contexts. Overall, we study has provided new information for understanding the mechanisms underlying oviparous ovarian development, which may help to improve artificial culture of an economically valuable penaeid shrimp species.

## Materials and Methods

### Animals

For molecular cloning, tissue distribution and ovarian development, healthy Pacific white shrimp (*L. vannamei*) were collected from the Haimao Shrimp Culture Center, (Zhanjiang, China) and they were maintained in filtered seawater [30 parts per thousand (ppt), pH 8.2] at 30°C under a 12-h dark:12-h light photoperiod with a feeding schedule of twice a day. The shrimp were anesthetized on ice and killed by decapitation. The animal experiments were conducted in accordance with the guidelines and approval of the ethics committees of South China Sea Institute of Oceanology, Chinese Academy of Sciences.

### Molecular Cloning of *Lv-VgR* cDNA

Total RNA was extracted from the ovaries of sexually mature female shrimp using TRIzol reagent (Invitrogen, Carlsbad, CA, United States) and reversely transcribed into first-strand cDNA with PrimeScript II^TM^ 1st strand cDNA Synthesis Kit (Takara, Dalian, China). Based on a unigene that was obtained from a *L. vannamei* Illumina transcriptome constructed by our lab previously and shares high sequence homology with the *P. monodon VgR*, gene-specific primers (Table 1) were designed to amplify four overlapped partial fragments of *VgR* in *L*. *vannamei* (*Lv-VgR*), and the corresponding full-length sequence was obtained by 5′- and 3′-rapid amplification of cDNA ends (RACE) as described previously (Chen et al., 2008).

**TABLE 1:** Primers used for cloning, RT-PCR, DIG-labeled DNA probe and the sequences for siRNA synthesis.

Primer name	Sequence (5′–3′)	Purpose
VgR-5′end-F VgR-5′end-R VgR-A-F VgR-A-R	GGAAGAAGCTCCTCCCAAGA AGTGGACGCAGTCTGTAACCT TGCGACAACGACGTGGATT TGTCGTTACACTTGACGGATTCAC	Primer for 5′ end fragment Primer for 5′ end fragment Middle fragment A Middle fragment A
VgR-B-F VgR-B-R VgR-C-F VgR-C-R	GTTCGCAAATCTTGTGGTCTGA CGGCATAAGGGCTTTCGTC TCACCTGCGGCAACAAGA CCCACCGTTTCCCAAGC	Middle fragment B Middle fragment B Middle fragment C Middle fragment C
VgR-D-F VgR-D-R VgR-3′race-F1 VgR-3′race-F2 VgR-3′race-F3 VgR-all-F VgR-all-R1	CTGACGAAAGCCCTTATGCCG TCGGCTGTGCTCACTATGGC TTCCTTGGACCCCGTTAGCA TTCTGTGATAATGGTCTGCGGGATG ATTGGACTAGCCCTGAGTGGAG GGAAGAAGCTCCTCCCAAGA TGCGTGCTTCACAAAACAGTTCA	Middle fragment D Middle fragment D 3′ RACE 3′ RACE 3′ RACE Full length Full length
VgR-all-R2 VgR-q-F VgR-q-R GAPDH-F GAPDH-R VgR-PF VgR-PR VgR-831-F VgR-831-R EGFP-482-F EGFP-482-R	CCACATTGATACCTGGATTTTACC TGGTTCTCCTCGTCTTGGCTCTG GGTGGTGGCGTTCGTGGTTG CCCTTCATCACGCTGGACTAC AACACACCAGTGGACTCAACGA TCACCTGCGGCAACAAGA CCCACCGTTTCCCAAGC GCGAAUCCCAAGACCAUUATT UAAUGGUCUUGGGAUUCGCTT GCAUCAAGGUGAACUUCAA UUGAAGUUCACCUUGAUGC	Full length RT-PCR RT-PCR RT-PCR RT-PCR Synthesis of DIG-labeled DNA probe Synthesis of DIG labeled DNA probe VgR siRNA VgR siRNA NC siRNA NC siRNA

### Bioinformatics Analysis

The gene organization of *Lv-VgR* was generated by comparing the obtained cDNA sequence in this study and the gene sequence which was obtained from the *L. vannamei* genome (Zhang X. J. et al., 2019). The open reading frame (ORF) of *Lv-VgR* was determined by ORF finder and the corresponding amino acid (a.a.) sequence was deduced by using ExPASy translate tool. The molecular weight and theoretical isoelectric point (pI) of Lv-VgR were calculated by ExPASy ProtParam tool. Signal peptide and transmembrane helices were predicted by SignalP 4.0 Server and TMHMM Server v.2.0, respectively. *N*- and *O*-linked glycosylation sites were identified by NetNGlyc 1.0 Server and YinOYang 1.2 Server, respectively. Structural domains were analyzed by using SMART program and ScanProsite program. Multiple a. a. sequences alignment was performed using Clustalx 1.8 and presented by GeneDoc. Phylogenetic tree was conducted by a Neighbor Joining method using MEGA6 with bootstrap of 1000 replicates.

### *Lv-VgR* Transcript in Different Tissues, Ovarian Developmental Stages, and Embryonic and Laval Stages

The tissue expression pattern of *Lv-VgR* mRNA were detected in selected tissues, included the heart, gill, eyestalk, intestine, thoracic nerve, ventral nerve, muscle and hepatopancreas from the sexually immature adult shrimp (7.85 ± 2.58 g), and sexually mature male (31.34 ± 5.36 g) and female shrimp (37.49 ± 6.91 g), respectively, and the testis from the sexually mature male shrimp and the ovary from the sexually mature female shrimp. The mRNA expression profile of *Lv-VgR* was further detected in the ovaries during maturation. In this case, previtellogenic female shrimp were selected for artificially induced maturation with unilateral eyestalk ablation and nutrition enhancement (Chen et al., 2018a), and ovarian development was defined into four stages, namely, the stages I–IV, based on the classification of predominant oocytes as described previously (Chen H. Y. et al., 2018). For ontogeny, the mRNA levels of *Lv-VgR* were detected in the embryonic and larval developmental stages included the zygote, blastula, gastrula, limb bud embryo, larva in membrane, nauplius, zoea, mysis, and post-larval. In this case, about thirty individuals were mixed together as a sample and three samples were detected in a developmental stage. The embryonic and larval samples were collected when 80% of the population had reached the objective stage, and the morphologies was determined as described previously (Wei et al., 2014). The tissue, embryonic and larval samples were immediately frozen in liquid nitrogen and stored in 80°C, or directly stored in RNAlater solution (Ambion, Austin, TX, United States) for further analysis. The transcript levels of *Lv-VgR* were measured by quantitative real-time PCR (qPCR). Briefly, total RNA was extracted with TRIzol reagent and reverse transcribed with PrimeScript^TM^ RT reagent Kit containing gDNA eraser (Takara). The cDNA samples obtained were then subjected to a Thermal Cycler Dice^®^ Real Time System III (Takara) for quantitative analysis with the TB Green^TM^ Premix EX Taq^TM^ II Kit (Takara) and specific qPCR primers for target genes (Table 1). In this case, *GAPDH* was used as an internal control based on its stable expression levels observed.

### *In situ* Hybridization

The cellular localization of *Lv-VgR* mRNA in the ovaries during different developmental stages were performed by *in situ* hybridization (ISH). Briefly, samples from the ovaries were removed quickly and fixed overnight in 4% Paraformaldehyde Fix Solution (BBI, Sangon Biotech, Shanghai, China) for overnight. After a series of concentrations of alcohol dehydration, and paraffin embedding, the ovarian samples were cut into 5-μm sections and mounted onto slides. The sections were then digested by Proteinase K (20 μg/ml) at 37°C for 25 min, prehybridized at 42°C for 2 h and hybridized with a digoxigenin (DIG)-labeled DNA probe (8 ng/μl, Roche, Basel, Switzerland, Table 1) against *Lv-VgR* mRNA at 42°C for overnight. The ISH signal was developed by a diaminobenzidine (DAB) method by incubation with horseradish peroxidase (HRP)-conjugated anti-DIG antibody, and the nucleus was restained with hematoxylin.

### Immunofluorescence

The antigens of Lv-VgR (a.a. 73–398) and Lv-Vg (a.a. 19–366) were generated by an *Escherichia coli* recombinant protein expression system. The polyclonal antibodies against VgR and Vg were purified from the serum of a rabbit with antigen injection of Freund’s adjuvant and 300 μg rVgR/rVg for four times at 12 days intervals (Genecreate Biological Engineering Company, Wuhan, China). Complete Freund’s adjuvant was used for the first immunization and incomplete Freund’s adjuvant were used for the three more immunizations. The VgR and Vg antibodies were labeled with the FITC dye (488 nm, Invitrogen) and Cy3 dye (532 nm, Invitrogen), respectively, for visualization of the endogenous proteins, and DAPI reagent (405 nm, Invitrogen) was used for staining of the cell nucleus. The immunofluorescence (IF) was performed as described previously (Pan et al., 2018), and fluorescent images were observed and recorded with a DM-IRB fluorescence microscopy (Leica, Frankfurt, Germany).

### RNA Interference for Inhibition of Ovarian Development

The roles of VgR in the ovarian development of female shrimp were investigated by using an RNA interference (RNAi) approach. Based on the *Lv-VgR* cDNA sequence, the potential small interfering RNA (siRNA) targeting sites were identified with siDirect version 2.0 and DSIR online program, and the specificity of potential siRNAs was assessed with a global Blast. In this study, EGFP-482 siRNA was used to be non-targeting siRNA (NC siRNA), and the *Lv-VgR* and NC siRNA were synthesized by Sangon Biotech Company (Shanghai, China).

Before injection of siRNA, 80 female shrimps with intact eyestalks were kept in a 15-m^3^ culture pond for nutrition enhancement for a week. After that, the shrimp were applied for unilateral eyestalk ablation and the ovaries began to develop subsequently. At 36 h later, 28 shrimp at the previtellogenic ovarian developmental stage were picked out and randomly transferred to seven tanks (4 individuals per 1-m^3^ tank). Shrimp in one tank were killed and sampled immediately for the 0 time point and in other six tanks were injected with siRNA as described before (Cai et al., 2017). Shrimp in each two tanks were set as one group and injected with 375 μl of DEPC-treated PBS, NC siRNA [200 ng/g body weight (bwt)] and *Lv-VgR* siRNA (200 ng/g bwt), respectively. The siRNA was diluted in DEPC-treated PBS. The shrimp in each group were killed and sampled at 24 and 48 h after injection. The GSIs were weighted and calculated after sampling, and the ovarian developmental stages were confirmed by the observation of paraffin sections with hematoxylin and eosin (H/E) staining. In parallel experiment, the efficiencies of *Lv-VgR* siRNA was assessed by qPCR as described above.

### Data Transformation and Statistical Analysis

For qPCR, the relative expression levels of *Lv-VgR* were calculated using the comparative Ct method with the formula 2^–Δ^ ^Δ^ ^*Ct*^. The raw data were simply transformed as a percentage of the mean values in the control group, and the statistical analysis were performed with SPSS (IBM Software, Seattle, WA, United States). For qPCR and GSI measurement, the data expressed as the mean ± SE (standard error) were analyzed by using Student’s *t*-test or one-way ANOVA followed by Fisher’s least significant difference (LSD) test.

## Results

### Molecular Cloning and Structural Characterization of *Lv-VgR*

**FIGURE 1:**
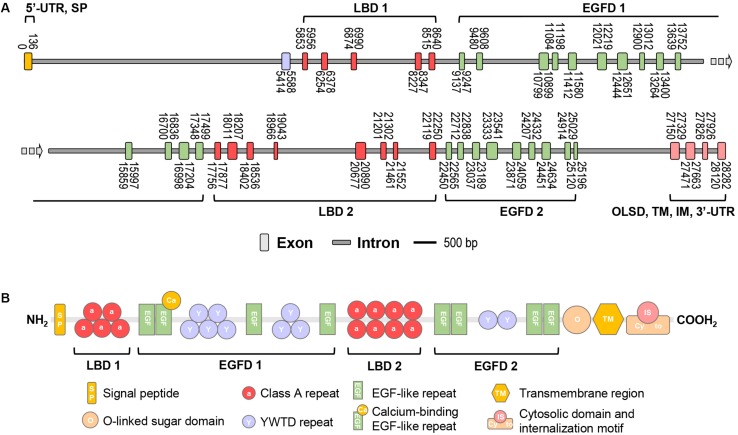
**(A)** The schematic diagram of exon/intron organization of *Lv-VgR* gene. Exons are marked by rectangle with orange, purple, red, green or pink, and introns are marked by rectangle with gray. The location of each exon and intron are labeled on the schematic diagram. The scale bar is 500 bp. **(B)** The structure domains of Lv-VgR protein, the signal peptide (SP), ligand-binding domain (LBD), EGF-precursor homology domain (EGFD), *O*-liked sugar domain (OLSD), transmembrane domain (TM) and cytosolic domain that contains internalization motif (IM) are indicated.

In this study, the full-length cDNA of vitellogenin receptor (*Lv-VgR*) was identified from the ovary of Pacific white shrimp. The *Lv-VgR* cDNA is 6019 bp in length, consisting of a 61-bp 5′ untranslated region (UTR), a 5832-bp ORF and a 126-bp 3′-UTR with a 28-bp poly-A tail (GenBank accession No. MN807241, Supplementary Figure S1). By screening the *L. vannamei* genomic data, the *Lv-VgR* gene was found to be located in the LOC113811783 of the LVANscaffold 2260, and the *Lv-VgR* gene is 6019 bp in length with 42 exons that were separated by 41 introns (Figure 1A).

The Lv-VgR precursor protein is 1943-amino acid (a.a.) in size, and it is composed of a 34-a.a. signal peptide and a 1909-a.a. mature protein with a deduced molecular weight of 213.58 kDa and pI of 5.21 (Supplementary Figure S1). Similar to the VgRs from other oviparous animals, Lv-VgR contains the five typical conserved modular elements for the LDLR superfamily (Figure 1B), namely, LBD, EGFD, OLSD, TM, and IM. Lv-VgR has two LBD and two EGFD. LBD 1 consist of five LDLR class A repeats and the LBD 2 consist of eight class A repeats. EGFD 1 contains eight YWTD repeats and four epidermal growth factor (EGF)-like repeats, while EGFD2 contains two YWTD repeats and four EGF-like repeats. A Thr- and Ser-rich OLSD are located in front of the TM region, followed by a cytosolic tail contains two IM motifs with the conversed NPX(Y/F) characteristics. Moreover, 10 putative *N*-linked glycosylation motifs and 2 putative *O*-linked glycosylation sites were observed in the Lv-VgR precursor.

### Comparative and Phylogenetic Analysis of *Lv-VgR*

**FIGURE 2:**
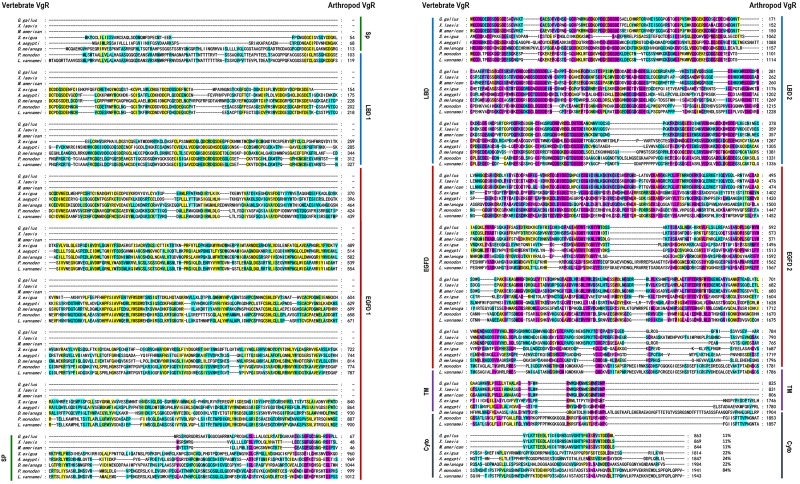
Amino acid sequence alignment of VgR from different oviparous animal, including *Gallus gallus* (P98165.1), *Xenopus laevis* (BAA22145.1), *Morone americana* (AAO92396.1), *Spodoptera exigua* (AOX13593.1), *Aedes aegypti* (AAK15810.1), *Drosophila melanogaster* (AAB60217.1), *Penaeus monodon* (ABW79798.1), and *Litopenaeus vannamei* (MN807241). The conserved amino acid residues are boxed in purple, yellow, and blue, respectively, based on the percentages of their identities. The sequence identities between Lv-VgR and the VgR from other species are also showed.

Multiple alignment was performed with the VgR a.a. sequences from various oviparous species (Figure 2). In this case, the newly obtained Lv-VgR only share high sequence identities of its counterpart in other crustaceans (*P. monodon*, 84%), but not other VgR from insects (*Aedes aegypti*, 24%; *Spodoptera exigua*, 22%; and *Drosophila melanogaster*, 22%) or vertebrates (*Gallus gallus*, 11%; *Xenopus laevis*, 11%; and *Morone americana*, 11%). The number of amino acid residues of VgRs from arthropods are almost twice of that from vertebrates, based on the fact that duplicated LBD/EGFDs are presented in the arthropod VgRs while only a single LBD/EGFD is found in the vertebrate VgRs. The VgR a.a. sequences were further analyzed comparatively by dividing into eight structural regions including SP, LBD 1, EGFD 1, LBD 2, EGFD 2, OLSD, TM, and cytoplasmic tail (Table 3). Although the identities for the whole sequences are low, the structural regions of VgRs are reasonably conserved between different species. The LBD/EGFD regions of vertebrate VgRs show relatively high conservation in both the former and latter LBD/EGFD regions (27–37%/15–18%) of the vertebrate VgRs, and are more similar to the latter one based on lengths and the identities of the a.a. sequence (Table 3).

**FIGURE 3:**
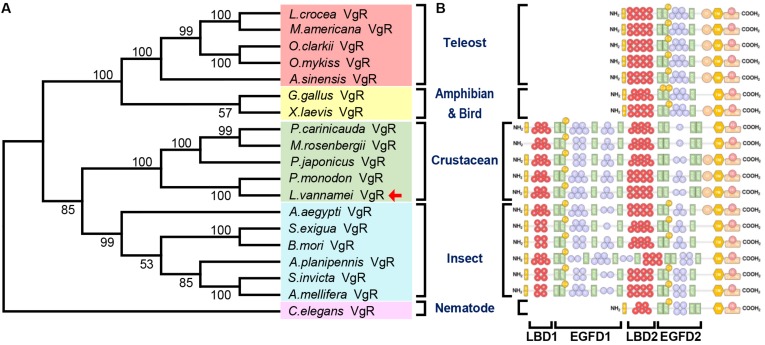
Phylogenetic analysis and protein structure comparison of VgRs in various oviparous species. **(A)** The phylogenetic tree was constructed by using neighbor-joining method with bootstrap value of 1,000. The amino acid sequences of VgR belonging to teleosts, amphibians and birds, crustaceans, insects and nematodes are marked with light red, yellow, green, blue, and pink, respectively. The Lv-VgR is marked with a red arrow following the taxon. The species name and corresponding GenBank accession numbers for the used sequences are listed in the Table 3. **(B)** The domain architecture on the right are correspondence with the species in the phylogenetic tree.

The Genbank accession numbers of protein used in this study are shown in Table 2. A phylogenetic analysis was conducted with the VgRs from multiple oviparous animal species (Figure 3A). The phylogenetic tree is divided into two three clusters, namely, the VgRs from vertebrates, arthropods and nematodes. The newly identified Lv-VgR is grouped into a sub-branch of crustacean VgRs that is closed to the branch of insect VgRs. The structure domains for the corresponding VgRs were also analyzed comparatively (Figure 3B). The characteristic domains, such as LBD, EGFD, OLSD, TM, and IM, are observed in most of LDLR superfamily members. However, the duplicated LBD/EGFD regions are only found in the arthropod VgRs but not in their counterparts from vertebrates and nematodes. In addition, OLSD is missing in a larger number of arthropod VgRs but remains in Lv-VgR.

**TABLE 2:** The Genbank accession numbers and the length of protein sequences used in this study.

Species	Gene	Genbank accession	Length	Type
*Gallus gallus*	VgR	P98165.1	863	Protein
*Xenopus laevis*	VgR	BAA22145.1	869	Protein
*Morone americana*	VgR	AAO92396.1	844	Protein
*Larimichthys crocea*	VgR	ASS77299.1	844	Protein
*Oncorhynchus mykiss*	VgR	CAD10640.1	847	Protein
*Oncorhynchus clarkii*	VgR	AHH55319.1	842	Protein
*Acipenser sinensis*	VgR	AWY62799.1	855	Protein
*Aedes aegypti*	VgR	AAK15810.1	1847	Protein
*Apis mellifera*	VgR	XP_026295652.1	1754	Protein
*Solenopsis invicta*	VgR	AAP92450.1	1782	Protein
*Bombyx mori*	VgR	ADK94452.1	1809	Protein
*Spodoptera exigua*	VgR	AOX13593.1	1814	Protein
*Agrilus planipennis*	VgR	XP_025835220.1	1863	Protein
*Penaeus monodon*	VgR	ABW79798.1	1941	Protein
*Pandalus japonicus*	VgR	AHL26192.1	1927	Protein
*Palaemon carinicauda*	VgR	AHB12420.1	1886	Protein
*Macrobrachium rosenbergii*	VgR	ADK55596.1	1889	Protein
*Caenorhabditis elegans*	VgR	AAD56241.1	925	Protein

**TABLE 3:** The identify between the Pacific white shrimp and other species in individual domain.

Region	SP			LBD 1				EGFD 1				LBD 2			
Special	Location	Length	Identity	Location	Length	Identity	Class A	Location	Length	Identity	YWTD + EGF	Location	Length	Identity	Class A
*G. gallus*	1–46	46	23%	50–375	326	28%	8	376–770	395	15%	5 + 3	50–375	326	37%	8
*X. laevis*	1–27	27	17%	31–356	326	30%	8	358–750	393	15%	5 + 3	31–356	326	36%	8
*M. americana*	1–25	25	13%	29–354	326	27%	8	356–749	394	16%	5 + 3	29–354	326	37%	8
*S. exigua*	1–17	17	8%	29–217	189	28%	4	218–938	725	21%	6 + 4	942–1283	342	32%	7
*A. aegypti*	1–30	30	19%	44–251	208	32%	5	253–951	699	27%	7 + 4	955–1301	347	34%	8
*D. melanogaster*	NA	NA	NA	89–306	218	31%	5	309–1026	718	25%	7 + 4	1030–1377	348	32%	8
*P. monodon*	1–20	20	44%	77–279	203	88%	5	280–978	699	87%	8 + 4	981–1327	347	80%	8
*L. vannamei*	1–34	34	100%	93–295	203	100%	4	296–991	696	100%	8 + 4	994–1332	339	100%	8

**Region**	**EGFD 2**				**OLSD**			**TM**			**Cyto**			**Total length**	**Overall identity**
**Special**	**Location**	**Length**	**Identity**	**YWTD** + **EGF**	**Location**	**Length**	**Identity**	**Location**	**Length**	**Identity**	**Location**	**Length**	**Identity**		

*G. gallus*	376–770	385	18%	5 + 3	NA	NA	NA	787–809	23	17%	810–863	54	12%	863	11%
*X. laevis*	358–750	393	18%	5 + 3	751–792	42	9%	793–815	23	26%	816–869	54	12%	869	11%
*M. americana*	356–749	394	18%	5 + 3	750–767	17	15%	768–790	23	17%	791–844	54	13%	844	11%
*S. exigua*	1284–1662	379	19%	3 + 3	NA	NA	NA	1689–1711	23	12%	1712–1814	103	11%	1814	22%
*A. aegypti*	1302–1689	388	20%	3 + 3	1690–1721	31	18%	1722–1744	23	16%	1745–1847	103	11%	1847	24%
*D. melanogaster*	1378–1770	393	16%	3 + 3	NA	NA	NA	1799–1820	22	37%	1821–1984	164	8%	1984	22%
*P. monodon*	1328–1769	442	81%	3 + 4	1770–1783	13	85%	1784–1806	23	86%	1807–1941	135	92%	1941	84%
*L. vannamei*	1333–1774	442	100%	2 + 4	1775–1787	12	100%	1788–1810	23	100%	1811–1943	133	100%	1943	100%

### Transcript Expression of *Lv-VgR* in Different Tissues, Ovarian Developmental Stages, and Embryonic and Laval Stages

**FIGURE 4:**
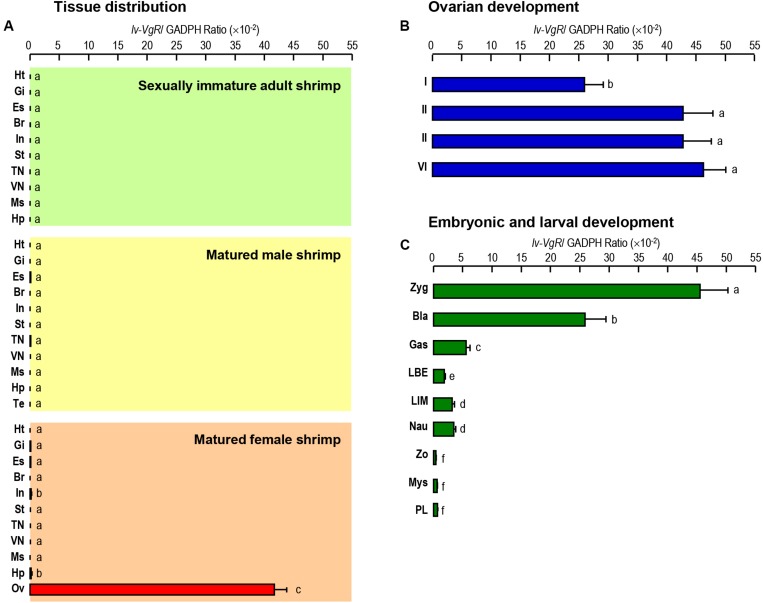
**(A)** Tissue distribution of *Lv-VgR* mRNA expression detected in different tissue including: heart (Ht), gill (Gi), eyestalk (Es), brain (Br), intestine (In), stomach (St), thoracic nerve (TN), ventral nerve (VN), muscle (Ms), hepatopancreas (Hp), testicle (Ts), and ovary (Ov). **(B)** The expression levels of *Lv-VgR* mRNA in different ovarian development stages (stages I–IV). **(C)** The expression levels of *Lv-VgR* mRNA observed in different embryonic and larval developmental periods including zygote (Zyg), blastula (Bla), gastrula (Gas), limb bud embryo (LBE), larva in membrane (LIM), nauplius (Nau), zoea (Zo), mysis (Mys), and post-larval (PL). In this study, the results are expressed as the mean ± SE (*n* = 3).

The transcript expression of *Lv-VgR* were detected in multiple tissues from sexually immature shrimp, and sexually mature male and female shrimp by quantitative qPCR. As shown in Figure 4A, the *Lv-VgR* mRNA was specifically expressed in the ovary of sexually mature female shrimp, but hardly detected in any other tissues form sexually immature and mature shrimp. During the ovarian development, the expression levels of Lv-VgR increased continuously from stage I to stage IV, and reached the highest abundance in the stage IV (Figure 4B). During the embryonic and larval developmental stages, the highest expression level of *Lv-VgR* was observed at the stage of zygote and reduced sharply at the stages of blastula and gastrula. The expression of *Lv-VgR* was further decreased at the stage of zoea and kept at very low levels in the larval (Figure 4C).

### *In situ* Hybridization for *Lv-VgR* mRNA During Ovary Development

**FIGURE 5:**
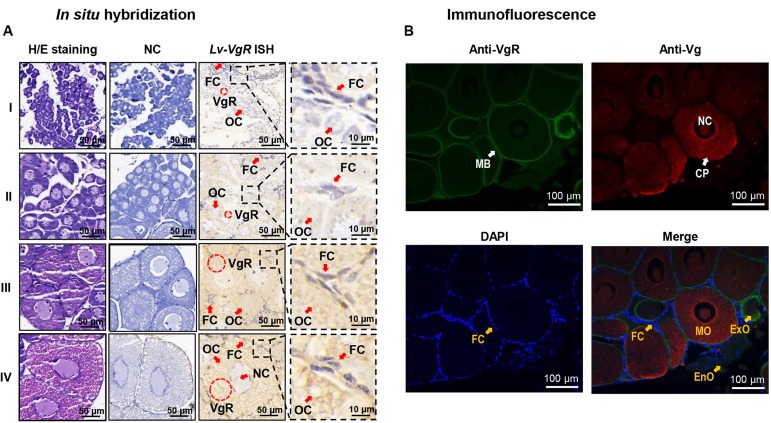
**(A)** *In situ* hybridization of *Lv-VgR* mRNA expressing cells in different ovarian stages. Ovarian developmental states were represented by I (previtellogenesis, GSI of 0.3–0.9%), II (primary vitellogenesis, GSI of 1.0–2.4%), III (secondary vitellogenesis, GSI of 2.5–5.0%), IV (maturation, GSI of >5.0%). The cell types and markers labeled include oocyte (Oc), follicle cell (Fc), nucleus (Nc), and the positive signal (*VgR* mRNA). Scale bars are 50 and 10 μm for the inserts. **(B)** Immunofluorescence for the cellular location of Vg and VgR protein in the ovary. The VgR protein, Vg protein and cell nucleus are stained by anti-VgR antibody with FITC (green), anti-Vg antibody with Cy3 (red), and DAPI (blue), respectively. The markers labels include plasma membrane (MB), cell nucleus (NC), cytoplasm (CP), endogenous vitellogenetic oocytes (EnO), exogenous vitellogenetic oocytes (ExO), mature oocytes (MO), and follicle cells (FC). The scale bars were 100 μm.

The cellular expression of *Lv-VgR* mRNA were detected by ISH in the ovaries in different developmental stages. Compared with the negative control group without digoxygenin probe, the experimental group showed strong positive signals for *Lv-VgR* expressed cells. As shown in Figure 5A, *Lv-VgR* mRNA was abundant in the cytoplasm of the oocytes. During development of the oocytes, the intensity of hybridization signals increased, reaching a maximum during stages III–IV, which is consistent with the mRNA expression profile by qPCR.

### Immunofluorescence for Localization of VgR and Vg Protein in the Ovary

By IF, the protein distribution of VgR and Vg were exhibited in the ovarian sections (Figure 5B). In this case, no signals of VgRs were presented in the follicle cells. In the endogenous vitellogenetic oocytes, immunopositive signals of VgRs were only detected in the cytoplasm of, and in the exogenous vitellogenetic oocytes, VgRs were shown to gather from the cytoplasm to plasma membrane. On the contrary, VgR were detected strongly in the plasma membrane and weakly in the cytoplasm of the mature oocytes. In parallel experiment, Vg was exhibited a evenly distribution in the cytoplasm of the exogenous vitellogenetic and mature oocytes.

### Inhibition of Ovarian Development by RNAi of Lv-VgR

**FIGURE 6:**
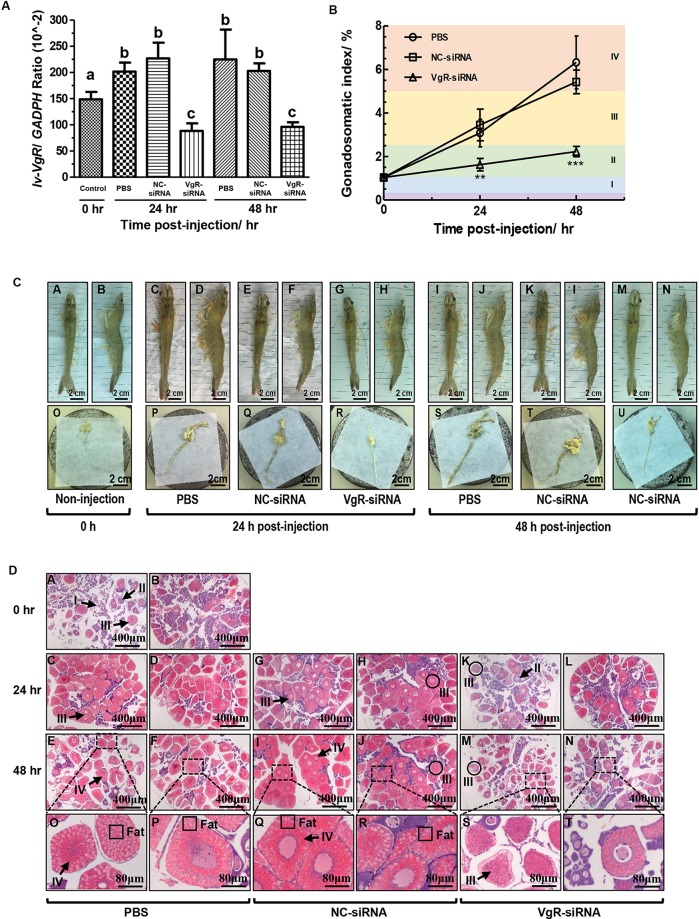
**(A)** The expression level of *Lv-VgR* mRNA at 0, 24, and 48 h after the injection of PBS, NC-siRNA or VgR-siRNA, and different lowercase letter represented a significant level (*P* < 0.001, ANOVA followed by Fisher’s LSD test). In this study, the data are expressed as mean ± SE (*n* = 6). **(B)** The gonadosomatic index (GSI) of female shrimp at 0, 24, and 48 h after the injection of PBS, NC-siRNA or VgR-siRNA. Different ovarian developmental stages were represented by I, II, III, and IV, respectively. The significant differences were assessed using Student’s *t*-test (*P < 0.05, **P < 0.01, ***P < 0.001). In this study, the data are expressed as mean ± SE (*n* = 4). **(C)** Morphological and anatomical analysis for the ovarian development of *L. vannamei*. Panels **(A,C,E,G,I,K,M)** are dorsal views. Panels **(B,D,F,H,J,L,N)** are lateral views. Panels **(O–U)** are the ovaries obtained from the shrimp above. Scale bars: 2.5 cm. **(D)** Sections of ovaries stained with hematoxylin and eosin. Panels **(A,B)** are uninjected controls, **(C–F)** saline, **(G–J)** NC-siRNA, **(K–N)** VgR-siRNA injected treatments, **(O–T)** are the enlarged regions above. Numerals I–IV refer to the developmental stages of the oocytes. Scale bars: 400 μm, inserts 8 μm.

Injection of VgR-siRNA can effectively reduce the transcript expression of *Lv-VgR* in the *L. vannamei* ovaries (Figure 6A). The effect of *Lv-VgR* mRNA silencing on ovarian development was assessed by measuring their GSIs (Figure 6B). In either the blank group injected with PBS or the negative group injected with NC-siRNA, the ovaries of shrimp kept increasing continuously, and reached the corresponding GSIs of stages III and IV at 24 and 48 h after injection, respectively. In contrast, the GSIs of shrimp injected with VgR-siRNA remained at stage II, which was much lower than those injected with PBS or NC-siRNA at both the 24 and 48 h after injection. In summary, silencing of *Lv-VgR* mRNA is effective in inhibiting ovarian development.

By morphological and anatomical observations, the ovaries from shrimp with different treatments have different features (Figure 6C). The ovaries from those without injection were thin and transparent in color of cyan [Figure 6C(O)], and hardly to be observed through the carapace by either dorsal or lateral views [Figures 6C(A,B)]. After that, the ovaries from those injected with PBS or NC-siRNA at 24 h became hypertrophic. Due to accumulation of Vg and other substances, such as carotenoid, the ovaries were became yellow in color and no longer transparent [Figures 6C(P,Q)]. Therefore, the ovaries can be clearly observed through the carapace at this time point [Figures 6C(C–F)]. Finally, the ovaries from those injected with PBS or NC-siRNA at 48 h reached their maximum volume and became fragile [Figures 6C(S,T)]. However, the ovaries form those injected with VgR-siRNA remained thin at either 24 or 48 h [Figures 6C(R,U)]. Interestingly, although the ovaries form the VgR-siRNA injected shrimp kept in the sizes similar to those at stage II, they could be observed through the carapace, since that their color changed to yellow and no longer transparent [Figures 6C(G,H,M,N)].

By further histological analysis with H/E staining (Figure 6D), the ovaries from the 0-h non-injected group mainly comprised of oogonia and previtellogenic oocytes, with a few endogenous vitellogenetic oocytes. The ovaries from the PBS and NC-siRNA injected groups were full of exogenous vitellogenetic oocytes at 24 h after injection, and exogenous vitellogenetic oocytes and mature oocytes with oil droplet deposition at 48 h after injection. On the contrary, the ovaries from the VgR-siRNA injected group still contained a large number of previtellogenic oocytes and endogenous vitellogenetic oocytes at 24 and 48 h after injection. It is notable that the sizes of the exogenous vitellogenetic oocytes found in the ovaries from the VgR-siRNA injected group were significantly smaller than those from the non-injected, and PBS and NC-siRNA injected groups, indicating that the accumulation of Vg was blocked by VgR silencing in the developing oocytes.

## Discussion

In this study, the full-length cDNA of *VgR* has been obtained from Pacific white shrimp *L. vannamei* (Supplementary Figure S1), with predictions of its corresponding gene structure (Figure 1A) and protein structural domains (Figure 1B). Similar to other members in the LDLR superfamily, Lv-VgR contains five high conserved regions including LBD with Class A repeats, EGFD with EGF-like and YWTD repeats, OSLD, TM, and IM (Figure 3B). The Class A, EGF-like and YWTD repeats present in almost all VgRs in oviparous animals, but the specific numbers for these repeats vary in different species (Figure 3B). In general, the LBD of vertebrate VgRs consist of 8 Class A repeats (Okabayashi et al., 1996; Hiramatsu et al., 2004), while the LBD 1 and LBD 2 of arthropod VgRs normally have 4–5 and 6–8 repeats, respectively (Schonbaum et al., 1995; Chen et al., 2004; Tiu et al., 2008; Lin et al., 2013). Similarly, there were 5 YWTD repeats surrounded by EGF-like repeats in the EGFD of vertebrate VgR (Bujo et al., 1994), and 7–9 and 1–4 repeats are shown in the EGFD 1 and EGFD 2 of arthropod VgRs (Chen et al., 2004; Tiu et al., 2008). It has been previously reported that EGFD indirectly affects ligand/receptor binding and dissociation (Davis et al., 1987; Russell et al., 1989), and that variation of the extracellular domains of VgRs reflect the type of potential ligands (Hiramatsu et al., 2015). For example, VgRs in Chicken can recognize Vg, VLDLR and Riboflavin binding protein (Stifani et al., 1990; Mac Lachlan et al., 1994). Similarly, the types and numbers of intracellular motifs as the internalized signals are not identical for different members in the LDLR family, e.g., for the crustacean VgRs, the sequences of IM are normally NP(X)F (Tiu et al., 2008; Roth and Khalaila, 2012; Bai et al., 2016), while for most of the insect VgRs, they are usually L(I/L) (Tufail and Takeda, 2009).

Phylogenetically, major yolk protein precursor (Vg) of crustacean is closely related to the insect apolipophorin II/I (ApoLp-II/I) and vertebrate apolipoprotein B (ApoB), but distant from the Vgs from other oviparous species (Avarre et al., 2007). It is speculated that in the lineage of crustacean, the true ortholog of ancestral *V*g gene has been lost, and this group of animals may then utilize either Apo and/or clotting protein (CP) as the major yolk protein (Wu et al., 2013). Thus, the ancestral *Apo* gene has been named as *Vg* in the crustacean lineage. However, there is still a lack of clarity regarding the generation of VgRs in different taxons of oviparous animals. Based on the phylogenetical analysis, our present study has shown that the ancestral arthropod VgR gene remains in both Insecta and Crustacea as the modern insect and crustacean VgR genes. Furthermore, there are two LBD/EGFD regions are present in the arthropod VgRs while only a single LBD/EGFD region is found in the vertebrate VgRs (Figure 3B). Combined with the phylogenetical and structural analyses, it is speculated that the LBD 1/EGFD 1 region is replicated from the LBD 2/EGFD 2 region, and this region duplication occurs after the differentiation of arthropod VgR and before the divergence of insects and crustaceans.

mRNA expression of *VgR* was specifically detected in the ovary of *L. vannamei* (Figure 4A), consistent with previous reports in vertebrates (Bujo et al., 1994; Okabayashi et al., 1996; Mizuta et al., 2013) and other crustaceans (Tiu et al., 2008). During ovarian development, expression of *Vg* in the hepatopancreas of *L. vannamei* increased continuously and reached a peak in stage III of ovarian development, reducing rapidly at stage IV (Chen et al., 2018b). The current study illustrated that the expression levels of *Lv-VgR* in the ovary increased continuously from the ovarian developmental stage-I to stage-IV (Figure 4B), indicating that a exogenous Vg absorption process similar to other oviparous animals which exhibit exogenous vitellogenesis, such as fish (Hiramatsu et al., 2015; Hara et al., 2016) and insects (Sappington and Raikhel, 1998). During embryonic and larval stages, the expression levels of *Lv-VgR* was reduced rapidly, in accordance with the trend of yolk consumption. After breaking out of the egg membrane, the nauplius still consumed the remaining yolk sac nutrients. The expression of *Lv-VgR* were detectable until the zoea begin feeding and subsequently no longer need the yolk nutrition.

The cellular localizations of *Lv-VgR* mRNA and protein were determined by ISH and IF, respectively. In this case, *Lv-VgR* mRNA is expressed in the oocytes but not the follicle cells of the ovary (Figure 5A), and Lv-VgR protein is located in the membrane of oocytes while the Lv-Vg protein accumulates densely in the cytoplasm of oocytes (Figure 5B). In fish, it is generally considered that Vg enters the growing oocytes via selective endocytosis mediated by the plasma membrane VgR (Hiramatsu et al., 2013). In the process of oocyte development, follicle cells perform many essential physiological functions including supplying glycogen, engulfing cell debris, promoting ovulation, and synthesis of steroid hormones (e.g., androgen, estrone and 17β-estradiol) and cellular transmitters (e.g., insulin-like growth factor, bone morphogenetic proteins and transforming growth factor ß) (Young and McNeilly, 2010; Chen et al., 2018a). Distribution of VgR mRNA/protein is absent in the follicle cells of the *L. vannamei* ovary, similar to that reported in *P. monodon* (Tiu et al., 2008). However, in *P. monodon*, VgR was distributed evenly throughout the oocyte cytoplasm (Tiu et al., 2008), which is likely due to the endocytic VgR being detected by IF in cytoplasm. During the ovarian development of *L. vannamei*, the VgR protein distributed from evenly in the cytoplasm to strongly located in the plasma membrane (Figure 5B). Meanwhile, the Vg protein started to be rapidly accumulated in the oocytes. This result indicates that VgR protein transferring from the cytoplasm to plasma membrane predicts the beginning of the endogenous vitellogenetic stage of *L. vannamei*, and it is also the beginning for a rapid accumulation of yolk in the ovary.

The effects of *VgR* silencing on shrimp ovarian development were measured by morphological, anatomical and histological methods. The stunting of ovarian development was significantly observed in the shrimp injected with VgR-siRNA. Based on the GSI values, the ovaries in VgR-siRNA treated shrimp remained in stage II until 48 h after the injection, showing the inhibitory effect of VgR blockage on ovarian development (Figure 6B). In general, features of ovaries will appear a series of changes during development, such as from cyan to yellow, from transparent to non-transparent, from thin to hypertrophic, from elastic to fragile. The ovaries from VgR-siRNA injected shrimp were thin and elastic but no longer transparent and yellow [Figures 6C(R,U)]. Remaining thin and elastic showed that the ovaries lacked Vg accumulation. Becoming non-transparent and yellow meant that the hydrolysis of existent Vg protein and the accumulation of carotenoids were not inhibited. By histological level, the sizes of the exogenous vitellogenetic oocytes found in the ovaries from VgR-siRNA injected shrimp [marked by black circles in Figures 6D(K,M)] were smaller than those in normal condition [Figures 6D(H,J)], showing that the nutritional deficiencies of oocytes by VgR blockage. As a whole, it is speculated that silencing of Lv-VgR can reduce the transcription and translation levels of Lv-VgR and resulted in a limitation of Vg accumulation but not other processes in ovarian development.

In summary, we have cloned the full-length cDNA sequence of vitellogenin receptor from the Pacific white shrimp, characterized its genomic organization and protein structural domains. A phylogenetical model of VgRs in different taxons of oviparous animals was proposed. Transcripts of *Lv-VgR* were found to be specifically expressed in the ovaries of female shrimps, with increasing expression levels during ovarian development, and rapidly declines during embryogenesis. In the ovary, VgR protein is located in the cell membrane of maturing oocytes while the accumulated Vg protein is evenly distributed in the cytoplasm. In this vein, we reason that transfer of VgR protein from the cytoplasm to plasma membrane predicts the beginning of rapid yolk accumulation in the ovary of *L. vannamei*. Silencing of *VgR* transcript expression by siRNA injection was effective in stunting ovarian development of *L. vannamei*. The mode of action of VgR was further investigated by observations at the morphological, anatomical and histological levels. While evidently important to the process of Vg accumulation, VgR signaling does not seem to impact other steps of ovarian development. This study has furnished new information for advancing our understanding on the mechanisms of ovarian development underlying crustacean reproduction biology.

## Data Availability Statement

All datasets generated for this study are included in the article/Supplementary Material.

## Ethics Statement

The animal experiments were conducted in accordance with the guidelines and approval of the Ethics Committees of South China Sea Institute of Oceanology, Chinese Academy of Sciences.

## Author Contributions

XuW, CH, and TC conceived and designed the experiments. YR, N-KW, XZ, XiW, and XJ performed the experiments. YR, N-KW, XZ, CZ, CR, PL, XJ, and TC analyzed the data. CZ, CR, XJ, JJ, XuW, CH, and TC contributed reagents, materials, and analysis tools. YR, N-KW, XuW, CH, and TC wrote the manuscript.

## Conflict of Interest

JJ was employed by the company Guangdong Haimao Investment Co., Ltd. The remaining authors declare that the research was conducted in the absence of any commercial or financial relationships that could be construed as a potential conflict of interest.

## Supplementary Material

FIGURE S1 Nucleotide and deduced amino acid sequences of *Lv-VgR* cDNA. The signal peptide (SP) is underlined with red line (a.a. 1–34). The ligand-binding repeats (LBDs) are a.a. 93–295 and a.a. 994–1332, and the Class A repeats are boxed in green. The EGFPs are a.a. 296–991 and a.a. 1333–1774, and the EGF, EGF-CA, YWTD repeats are boxed in light blue, dark green and purple, respectively. The O-liked sugar domain (OLSD) are a.a. 1778–1787 underlined with dark point line and the transmembrane region (TM) are a.a. 1778–1810 boxed in yellow. The putative *N*-linked glycosylation sites (NLGS) are underlined with dark straight line. The internalization motif (IM) is boxed in gray.
