# Supplementary material for: Vitellogenin Receptor (VgR) Mediates Oocyte Maturation and Ovarian Development in the Pacific White Shrimp (Litopenaeus vannamei)
Source: Front Physiol. 2020 May 15;11:485. doi: 10.3389/fphys.2020.00485 (PMC7243368; doi:10.3389/fphys.2020.00485)
Supplement: FIGURE S1 — Nucleotide and deduced amino acid sequences of Lv-VgR cDNA. The signal peptide (SP) is underlined with red line (a.a. 1–34). The ligand-binding repeats (LBDs) are a.a. 93–295 and a.a. 994–1332, and the Class A repeats are boxed in green. The EGFPs are a.a. 296–991 and a.a. 1333–1774, and the EGF, EGF-CA, YWTD repeats are boxed in light blue, dark green and purple, respectively. The O-liked sugar domain (OLSD) are a.a. 1778–1787 underlined with dark point line and the transmembrane region (TM) are a.a. 1778–1810 boxed in yellow. The putative N-linked glycosylation sites (NLGS) are underlined with dark straight line. The internalization motif (IM) is boxed in gray. [file Data_Sheet_1.PDF]

Supplement. 1 Nucleotide and deduced amino acid sequences of lv-VgR cDNA

```
1    ggaagaagctcctcccaagattgggtcgtcgtgaaaatcctcgaacagtcgaggcagcgac
62   atgacagcgcagggacgtacgacggcggcggtcgggtccagcatgttgccttggagggtggttctcctcgtcttggctctgcacgcgggg
1    M  T  A  Q  G  R  T  T  A  A  V  G  S  S  M  L  P  W  R  V  V  L  L  V  L  A  L  H  A  G
152  gcatctcggggacaaggagatgatgccaacagcgtccgttcccggatgatgcgccccttccggaatgtcgacagcgcccccaacgccaca
31   A  S  R  G  Q  G  D  D  A  N  S  V  R  S  R  M  M  R  P  F  R  N  V  D  S  A  P  N  A  T
242  accacgaacgccaccaccaccaccaccaccagagctacaagtagtgttcaagttccggttaccacagaagttcctttgacagatgaagcg
61   T  T  N  A  T  T  T  T  T  T  R  A  T  S  S  V  Q  V  P  V  T  T  E  V  P  L  T  D  E  A
332  acccctgcctgccagccgccgcagtttgcgtgtggcggcagctcggggacctgcttgcagtcctcgcagcgctgtgacggcttctctgac
91   T  P  A  C  Q  P  P  Q  F  A  C  G  G  S  S  G  T  C  L  Q  S  S  Q  R  C  D  G  F  S  D
422  tgcccggatgggtcagacgagcacaactgcaacgtgtgcgacgagagccggatgttccgctgcctgaacggccaatgcatcaccaagttc
121  C  P  D  G  S  D  E  H  N  C  N  V  C  D  E  S  R  M  F  R  C  L  N  G  Q  C  I  T  K  F
512  tggcgatgcgacaacgacgtggattgcttcgaccacagcgacgagcagggatgcccggccaccgaggaaagcgcctgtccgactggcttc
151  W  R  C  D  N  D  V  D  C  F  D  H  S  D  E  Q  G  C  P  A  T  E  E  S  A  C  P  T  G  F
602  ttcgagtgcacctcaggcgagtgcgtgccgaggtcgtgggtctgcgacggggagagcgactgcaccgaccacacggatgagaccaagtgc
181  F  E  C  T  S  G  E  C  V  P  R  S  W  V  C  D  G  E  S  D  C  T  D  H  T  D  E  T  K  C
692  tcgtctaccccgtgacctgtcagccacaccagttcagatgcagaaccggtacctgtattgctgccgccttcaggtgtgatggggaactg
211  S  S  T  P  V  T  C  Q  P  H  Q  F  R  C  R  T  G  T  C  I  A  A  A  F  R  C  D  G  E  L
782  gactgtcccagtggcgaagacgaggcccactgcagtgatatcaagtgcagcgaatcccaagaccattaccagtgcaagagcggcgaatgc
241  D  C  P  S  G  E  D  E  A  H  C  S  D  I  K  C  S  E  S  Q  D  H  Y  Q  C  K  S  G  E  C
872  atttccattcaagcggtttgcgaccagcacgaagactgccgggatggtagcgacgaggaaggcctttgctcggagacgtgtaaggttaca
271  I  S  I  Q  A  V  C  D  Q  H  E  D  C  R  D  G  S  D  E  E  G  L  C  S  E  T  C  K  V  T
962  gactgcgtccacctgtgcaaaatgaccccacagggaccccactgtatgtgcaaagaaggctatgcaagaaatacgtctaatggttgtata
301  D  C  V  H  L  C  K  M  T  P  Q  G  P  H  C  M  C  K  E  G  Y  A  R  N  T  S  N  G  C  I
1052 gatgtgaacgagtgtgctgccaacttctcagtctgtgatcacttctgccaaaacatggacggcggcttcaattgctcctgtgctgaggac
331  D  V  N  E  C  A  A  N  F  S  V  C  D  H  F  C  Q  N  M  D  G  G  F  N  C  S  C  A  E  D
1142 tatatgttgcaggctgacgggaaatcctgcaaaacgacagtacgaggcgacgctctactttcatggcacaaggaaaggacataaggatg
361  Y  M  L  Q  A  D  G  K  S  C  K  T  T  V  R  G  D  A  L  L  F  M  A  Q  G  K  D  I  R  M
1232 ttggatcttggccttacgctctactcgcagatctattcaggcttctctcagagtatagccattgcgtatgacccggtcgatgacatggtc
391  L  D  L  G  L  T  L  Y  S  Q  I  Y  S  G  F  S  Q  S  I  A  I  A  Y  D  P  V  D  D  M  V
1322 tactggagcaccgatcagggagtcttcaggatatctcgaagtggaggcacgatgccgtctatcgtagtcaacgaaggcgtgggcatggtc
421  Y  W  S  T  D  Q  G  V  F  R  I  S  R  S  G  G  T  M  P  S  I  V  V  N  E  G  V  G  M  V
1412 gaagggctcgccgtcgactggctcgggaggaatctgtacatgacggactcgatgctcaagcagataatggtgtgctccctcagcggaacc
451  E  G  L  A  V  D  W  L  G  R  N  L  Y  M  T  D  S  M  L  K  Q  I  M  V  C  S  L  S  G  T
1502 tcctgccacgtgctgctgtcggacctcacgcacccgagaggcatacagctggacttggagaacagatacgtgtactggactgacgtaaac
481  S  C  H  V  L  L  S  D  L  T  H  P  R  G  I  Q  L  D  L  E  N  R  Y  V  Y  W  T  D  V  N
1592 cagtcgaccttggagcgagctggcctggatgggttacgtcgaacagtgctcatttcggacggcgtgaggtggcccaatgggctctggata
511  Q  S  T  L  E  R  A  G  L  D  G  L  R  R  T  V  L  I  S  D  G  V  R  W  P  N  G  L  W  I
1682 gatgctccagcacgaaggatatacatagcggatgcccataccaacgaggtgttccatgtgaactataatggaccgatcgaaagtaccta
541  D  A  P  A  R  R  I  Y  I  A  D  A  H  T  N  E  V  F  H  V  N  Y  N  G  T  D  R  K  Y  L
1772 gcggaggcggcggtggaccacccgttcgccatcgccgtgtggcaggagcgcctgtactggagcgactgggagcacgaccacatccggtct
571  A  E  A  A  V  D  H  P  F  A  I  A  V  W  Q  E  R  L  Y  W  S  D  W  E  H  D  H  I  R  S
1862 tgtctgaagcgcactggcaagcagacgaagctccttgtcaagggaacccacaacaacttctttggactggctctctatcaccctgcactg
601  C  L  K  R  T  G  K  Q  T  K  L  L  V  K  G  T  H  N  N  F  F  G  L  A  L  Y  H  P  A  L
1952 atgccgctgatcgacaacccctgctcgttccgccagtgtagccacctgtgcttgctgtccccggctagtagatacacctgtgtgtgtcca
631  M  P  L  I  D  N  P  C  S  F  R  Q  C  S  H  L  C  L  L  S  P  A  S  R  Y  T  C  V  C  P
2042 gccgagatggagctggcgtccgataagcacacttgcatcgatctccccagacgcacatacccctttatcgccgacggaagcaagatcttc
661  A  E  M  E  L  A  S  D  K  H  T  C  I  D  L  P  R  R  T  Y  P  F  I  A  D  G  S  K  I  F
2132 cagctgtcccccgcctccacggccacagcaccttcgccgcttggacgcccggggtcaccctcaagcgcattggaggttttgcctacgac
691  Q  L  S  P  R  L  H  G  H  S  T  F  A  A  W  T  P  G  V  T  L  K  R  I  G  G  F  A  Y  D
2222 cccattcaggacactgtaattgtgagcgacatttgggaagggagcatctacagtgtcaacagggagacaggagtcacggtgcccattgta
721  P  I  Q  D  T  V  I  V  S  D  I  W  E  G  S  I  Y  S  V  N  R  E  T  G  V  T  V  P  I  V
2312 ccgggcatatctcgggcagttagcgtggctgtcgactggctacggaggaacgtgtactggatcgacggcacgaaggcggccgtggaggtg
751  P  G  I  S  R  A  V  S  V  A  V  D  W  L  R  R  N  V  Y  W  I  D  G  T  K  A  A  V  E  V
2402 atacgcgaggacggcgccttccgcacggagctcctgaaggcgatgccgcacctgacgagcatcaccttagccccctcctcgggttcatg
781  I  R  E  D  G  A  F  R  T  E  L  L  K  A  M  P  H  L  T  S  I  T  L  A  P  L  L  G  F  M
```

```
2492 tacgtgagcgacgccagtattgagccgttcatcatgcggtgtggtttggacgccaaatcctgcagcaagatcgtcacggtcgacctcgtc
811  Y V S D A S I E P F I M R C G L D A K S C S K I V T V D L V
2582 cagcccttgtcgattacctttgagacgaatcccgatatcaaacgcctgtactggtgtgaccgcgccctgggtcgtatcgagagtgtggcc
841  Q P L S I T F E T N P D I K R L Y W C D R A L G R I E S V A
2672 gaagacgggaccgaccgacgggtcttcgtgcaaaacgcgaagagccccgtttcggtcctggtgactcgttcgcaaatcttgtggtctgaa
871  E D G T D R R V F V Q N A K S P V S V L V T R S Q I L W S E
2762 gaacggacttctctcatctacgccgcttccaaattggataatagttctgtgcgggcaatggctcttgaaatgggcattccggaaaacgga
901  E R T S L I Y A A S K L D N S S V R A M A L E M G I P E N G
2852 gaacgttccctgaagctcatggaagttggatggaaagttcccgagcaactagcagcgacaaaccacccttgcctgcagagtaatgagagc
931  E R S L K L M E V G W K V P E Q L A A T N H P C L Q S N E S
2942 tgtagtcagttgtgccttggagacaactttagcgaaaaggtgtgcgcttgcagtttcggctacaaactccaggtggacttgagaacgtgt
961  C S Q L C L G D N F S E K V C A C S F G Y K L Q V D L R T C
3032 gaatccgtcaagtgtaacgacatccagtttcattgtttccgatcgcacacatgtattcctagttcctggaaatgtgacttgacccccgac
991  E S V K C N D I Q F H C F R S H T C I P S S W K C D L T P D
3122 tgccaagacggcgaagatgaggaggattgtaatcagccacaagcgtgtaaggagaaagagttccagtgttctacaggctcgtgcattaac
1021 C Q D G E D E E D C N Q P Q A C K E K E F Q C S T G S C I N
3212 aagctgtggacatgtgacggtgtgcacgactgcgaggatggctctgacgagaaactcgatgaatgctcgaacgtaacgtgtagcagcgtg
1051 K L W T C D G V H D C E D G S D E K L D E C S N V T C S S V
3302 cactggagatgcaagtcgggcatgtgcattccgaagatgtgggtttgtgatcaagagaaggaatgtgacgacggctcagacgagacggag
1081 H W R C K S G M C I P K M W V C D Q E K E C D D G S D E T E
3392 tgtgatacttcttgcccggaacacaaagtcgcttgtagagatggaaaatgtgtaccaaaggtgtggaaatgtgacggcgacaaagactgc
1111 C D T S C P E H K V A C R D G K C V P K V W K C D G D K D C
3482 ctggacggaagtgatgaggagaattgtccaactgagtgtaagagcaacgagttcacctgcggcaacaagaactgtgtgcccctccgagcc
1141 L D G S D E E N C P T E C K S N E F T C G N K N C V P L R A
3572 acctgtgacggcgaggacgactgtggcgacggctccgatgagggcctgccttcgtgccagcccccagccccccctccgacgtgccacaaa
1171 T C D G E D D C G D G S D E G L P S C Q P P A P P P T C H K
3662 ggccagatcatgtgcgagcgccacgacctgtcatccccgcccatctgcataccacttgttagcgtatgcaacggcgttcgggactgcccc
1201 G Q I M C E R H D L S S P P I C I P L V S V C N G V R D C P
3752 cttggcgaagacgaggactgtgattactgcgcccgccacgagttcagctgctcctcgcacggctgcattccgagagggtggatgtgtgac
1231 L G E D E D C D Y C A R H E F S C S S H G C I P R G W M C D
3842 ggggaaaaggactgcactgacggctctgacgaaagcccttatgccggttgtccgcctggcaatgacaccgttagtgatgccccgccgccg
1261 G E K D C T D G S D E S P Y A G C P P G N D T V S D A P P P
3932 ccgccgccagtctgtggaacgcacgagttcgagtgcgggagtggcggctgcatagcgtcgcgtcttgtgtgtgatggcttggtggactgt
1291 P P P V C G T H E F E C G S G G C I A S R L V C D G L V D C
4022 ctcgatggctccgatgagggcagcttgtgcgcaaaaacctgcttgggaaacggtgggtgtcaacatgtgtgtaaggaaggtccaaaaaat
1321 L D G S D E G S L C A K T C L G N G G C Q H V C K E G P K N
4112 cgcatttgctcttgttggaagggattcaaactcgccgaggatcagactagctgcgtagatgtgaaggaatgcgacgacgaggccacctgc
1351 R I C S C W K G F K L A E D Q T S C V D V K E C D D E A T C
4202 agccagaagtgtgaggaaagacatggctaccacttgtgctcctgcttacccggctatactcttagacaggatcaacgttcctgcaaacca
1381 S Q K C E E R H G Y H L C S C L P G Y T L R Q D Q R S C K P
4292 ataggcggcgacgaatatgtggtggcggtgcatcctgggtccatcctgaacatgtcccactccttccatctcgttgagaaagtgacgatg
1411 I G G D E Y V V A V H P G S I L N M S H S F H L V E K V T M
4382 ccgtctcatgttcagttttcgtctctcgagtttgcaccagagtcccatcattttgtttatgctgataaagctcatggaactattgggaag
1441 P S H V Q F S S L E F A P E S H H F V Y A D K A H G T I G K
4472 atgagcatggatggcaagctgaccacactccttaagcacagaaagcgccctcagggtctttccttggaccccgttagcaacagcgtctat
1471 M S M D G K L T T L L K H R K R P Q G L S L D P V S N S V Y
4562 ttctctgaagaattcggtaaagctgaagttgtggacaacggtttgccaagagcgcgcagggagacgagtgctgacggggcttattctgtg
1501 F S E E F G K A E V V D N G L P R A R R E T S A D G A Y S V
4652 ataatggtctgcgggatggacggcgacagagaatgcagcatggtgtaccaaggacacggtgaagagatccctgcaatccgcgtggcccca
1531 I M V C G M D G D R E C S M V Y Q G H G E E I P A I R V A P
4742 atatcaagacgactcttcttctgcacaaacaatatggcgcaggaagaggcaaaaattttcacctctgatatggatggcacatcggctaga
1561 I S R R L F F C T N N M A Q E E A K I F T S D M D G T S A R
4832 attatcagtcataaggttgtgaagtgcggggacctggcagtggatgaggccaaggagcgagtgtactggaccgatctttcccgtaatgtt
1591 I I S H K V V K C G D L A V D E A K E R V Y W T D L S R N V
4922 gtcgagtctgtcaaatggtcgggcgatggccaccggattataaaagaaaatgtacacacgccaattggactagccctgagtggagactgg
1621 V E S V K W S G D G H R I I K E N V H T P I G L A L S G D W
5012 gtgctgtggttggacacgcacaagcatcaggtaataaagtgcaacaaattcgatgtaggcgtctgcgagcagcacaccatgggctctgct
1651 V L W L D T H K H Q V I K C N K F D V G V C E Q H T M G S A
```

```
5102 ggcatagctttgattgttcagcatcgtttaagaatggagagttcaatgattggagcctgcacagcaaagaactgcagtcaccattgcatg
1681  G  I  A  L  I  V  Q  H  R  L  R  M  E  S  S  M  I  G  A  C  T  A  K  N  C  S  H  H  C  M
5192 atccaaatggacaaaaaggccaattgtatgtgcaaagtcggctacattacagcacccaaccgtcctaacgagtgtgtcaggttgaaatcg
1711  I  Q  M  D  K  K  A  N  C  M  C  K  V  G  Y  I  T  A  P  N  R  P  N  E  C  V  R  L  K  S
5282 tgtgaccacagcccttgtcgaggcgaaggtatttgcgaatcccactccgacacagagtttatatgcaggtgtcctgtagaccatgaaggg
1741  C  D  H  S  P  C  R  G  E  G  I  C  E  S  H  S  D  T  E  F  I  C  R  C  P  V  D  H  E  G
5372 gccttgtgcgaggtggctaagacgcccacagcagacaacagtgacagtagcagcgcaaccttaggcgtgtgcctcttcctgataatcttc
1771  A  L  C  E  V  A  K  T  P  T  A  D  N  S  D  S  S  S  A  T  L  G  V  C  L  F  L  I  I  F
5462 ggtgccctcattttgggctttattggtatcgcaagcgaccgttccccttttggaagggaaaaggagggcagcttcgcaagagatgcttt
1801  G  A  L  I  F  G  L  Y  W  Y  R  K  R  P  F  P  F  W  K  G  K  G  G  Q  L  R  K  R  C  F
5552 aaagcgaatcagacgctacgcttcgctaacccaggcttcggtattatttcccccaccactgtgcccaacggaaacacagcgtccggtatt
1831  K  A  N  Q  T  L  R  F  A  N  P  G  F  G  I  I  S  P  T  T  V  P  N  G  N  T  A  S  G  I
5642 aataccatcccctcaactccgcctgtcttgcgaggctctcacaacttcgaaaccctttctttaaaactgatgagcacgtgccggacacg
1861  N  T  I  P  S  T  P  P  V  L  R  G  S  H  N  F  E  N  P  F  F  K  T  D  E  H  V  P  D  T
5732 agtgcagactcagccatagtgagcacagccgactcgacctccatcaacatcgctcctcatcaggtggatctgacgacaccacagcacgta
1891  S  A  D  S  A  I  V  S  T  A  D  S  T  S  I  N  I  A  P  H  Q  V  D  L  T  T  P  Q  H  V
5822 ttgaagccaccggcagagaagaaggtcgagtgggacctctctcctttccaacccttgcagcctccggtgtgaaacaatgaattagaatac
1921  L  K  P  P  A  E  K  K  V  E  W  D  L  S  P  F  Q  P  L  Q  P  P  V  *
5912 atatactgatatagataaacttgttgtaattgttaaatgtttaatattataggataatatatatttctaaacctaaacgtaaaaaaaaa
6002 aaaaaaaaaaaaaaaaaa
```

Supplement. 1. Nucleotide and deduced amino acid sequences of lv-VgR cDNA. The signal peptide (SP) is underlined with red straight line (AA 1-34). The ligand-binding repeats (LBDs) are AA 93-295 and AA 994-1332, and the Class A repeats are boxed in green. The EGFPs are AA 296-991 and AA 1333-1774, and the EGF, EGF-CA, TWTD repeats are boxed in light blue, dark green and purple, respectively. The O-liked sugar domain (OLSD) are AA 1778-1787 underlined with dark point line and the transmembrane region (TM) are AA 1778-1810 boxed in yellow. The putative N-linked glycosylation sites (NLGS) are underlined with dark straight line. The internalization motif (IM) are boxed in gray.

SP
Signal peptide

Class A
Low-density lipoprotein receptor domain class A

EGF
Epidermal growth factor-like domain

EGF-CA
Calcium-binding EGF-like domain

YWTD
Low-density lipoprotein-receptor YWTD domain

TM
transmembrane region

NLGS
N-linked glycosylation site

IM
internalization motif

OLSD
O-liked sugar domain
